# Decoding per- and polyfluoroalkyl substances (PFAS) in hepatocellular carcinoma: a multi-omics and computational toxicology approach

**DOI:** 10.1186/s12967-025-06517-z

**Published:** 2025-05-02

**Authors:** Yanggang Hong, Deqi Wang, Zeyu Liu, Yuxin Chen, Yi Wang, Jiajun Li

**Affiliations:** 1 The Second School of Clinical Medicine, Wenzhou Medical University, Wenzhou, 325035 Zhejiang China; 2 The First School of Clinical Medicine, Wenzhou Medical University, Wenzhou, 325035 Zhejiang China

**Keywords:** PFAS, Network toxicology, Hepatocellular carcinoma, Machine learning, Single-cell sequencing, Molecular docking

## Abstract

**Background:**

Per- and polyfluoroalkyl substances (PFAS), particularly perfluorooctanoic acid (PFOA) and perfluorooctane sulfonate (PFOS), are synthetic chemicals known for their widespread use and environmental persistence. These compounds have been increasingly linked to hepatotoxicity and the development of hepatocellular carcinoma (HCC). However, the molecular mechanisms by which PFAS contribute to HCC remain underexplored.

**Methods:**

This study employs a multi-omics approach that combines network toxicology, integrated machine learning, single-cell RNA sequencing, spatial transcriptomics, experimental validation, and molecular docking simulations to uncover the mechanisms through which PFAS exposure drives HCC. We analyzed publicly available transcriptomic data from several HCC cohorts and used differential gene expression analysis to identify targets associated with both PFAS exposure and HCC. We constructed a protein–protein interaction (PPI) network and a survival risk model, the PFAS-related HCC signature (PFASRHSig), based on integrated machine learning to identify prognostic biomarkers, with the goal of identifying core targets of PFAS in HCC progression and prognosis. RT-qPCR and immunohistochemical (IHC) staining were used to validate the expression levels of the targets in both tumor and normal tissues. Molecular docking simulations were conducted to assess the binding affinities between PFAS compounds and selected target proteins.

**Results:**

Functional enrichment studies revealed that PFAS targets were associated with metabolic signaling pathways, which are actively involved in lipid, glucose, drug metabolism, etc. Through integrated machine learning and PPI network analysis, we identified six genes, APOA1, ESR1, IGF1, PPARGC1A, SERPINE1, and PON1, that serve as core targets of PFAS in both HCC progression and prognosis. These targets were further validated via bulk RNA-seq, single-cell RNA-seq, and spatial transcriptomics, which revealed differential expression patterns across various cell types in the HCC tumor microenvironment. The results of RT-qPCR and IHC staining were consistent with the in silico findings. Molecular docking simulations revealed strong binding affinities between PFAS compounds and these core targets, supporting their potential roles in PFAS-induced hepatocarcinogenesis.

**Conclusions:**

Our study highlights key molecular targets and pathways involved in PFAS-induced liver carcinogenesis and proposes a robust survival risk model (PFASRHSig) for HCC. These findings provide new insights into PFAS toxicity mechanisms and offer potential therapeutic targets for mitigating the health risks associated with PFAS exposure. Collectively, our findings help in advancing clinical applications by providing insights into disease mechanisms and potential therapeutic interventions.

**Supplementary Information:**

The online version contains supplementary material available at 10.1186/s12967-025-06517-z.

## Introduction

Hepatocellular carcinoma (HCC) is the most prevalent primary liver malignancy and the third leading cause of cancer-related mortality worldwide [1–3]. Over the past few decades, its incidence has significantly increased, driven by major risk factors such as chronic hepatitis B and C infections, non-alcoholic fatty liver disease (NAFLD), and excessive alcohol consumption [4]. Despite advances in diagnosis and treatment, the prognosis for HCC patients remains poor, with a five-year survival rate of less than 20% in most regions [5, 6]. The aggressive nature of HCC, coupled with its frequent late-stage diagnosis, underscores the urgent need for early detection strategies and a deeper understanding of its molecular mechanisms [7, 8].

The etiology of HCC is multifactorial and involves complex interactions among genetic predispositions, environmental toxins, and metabolic dysregulation. Among these, per- and polyfluoroalkyl substances (PFAS), a class of synthetic chemicals widely used in industrial and consumer products, are emerging as significant environmental carcinogens with potential hepatotoxic effects [9, 10]. PFAS are extensively found in non-stick cookware, water-repellent fabrics, firefighting foams, and food packaging, leading to widespread contamination of water and food sources [11]. Owing to their long environmental half-life and bioaccumulation properties, PFAS persist in human tissues, particularly in the liver, where they may disrupt metabolic homeostasis and induce carcinogenic effects [12, 13].

Epidemiological and preclinical studies have increasingly suggested a link between PFAS exposure and liver dysfunction, including hepatotoxicity and carcinogenesis [14, 15]. Some studies have reported elevated levels of PFAS in HCC patients, whereas others have highlighted their role in altering key metabolic pathways [16, 17]. However, existing research has several limitations: (i) most epidemiological studies rely on liver enzyme markers rather than direct histopathological evidence, (ii) preclinical studies often use high-dose PFAS exposure models that may not reflect real-world human exposure, and (iii) the molecular mechanisms linking PFAS to HCC remain largely unclear [18].

**Fig. 1:**
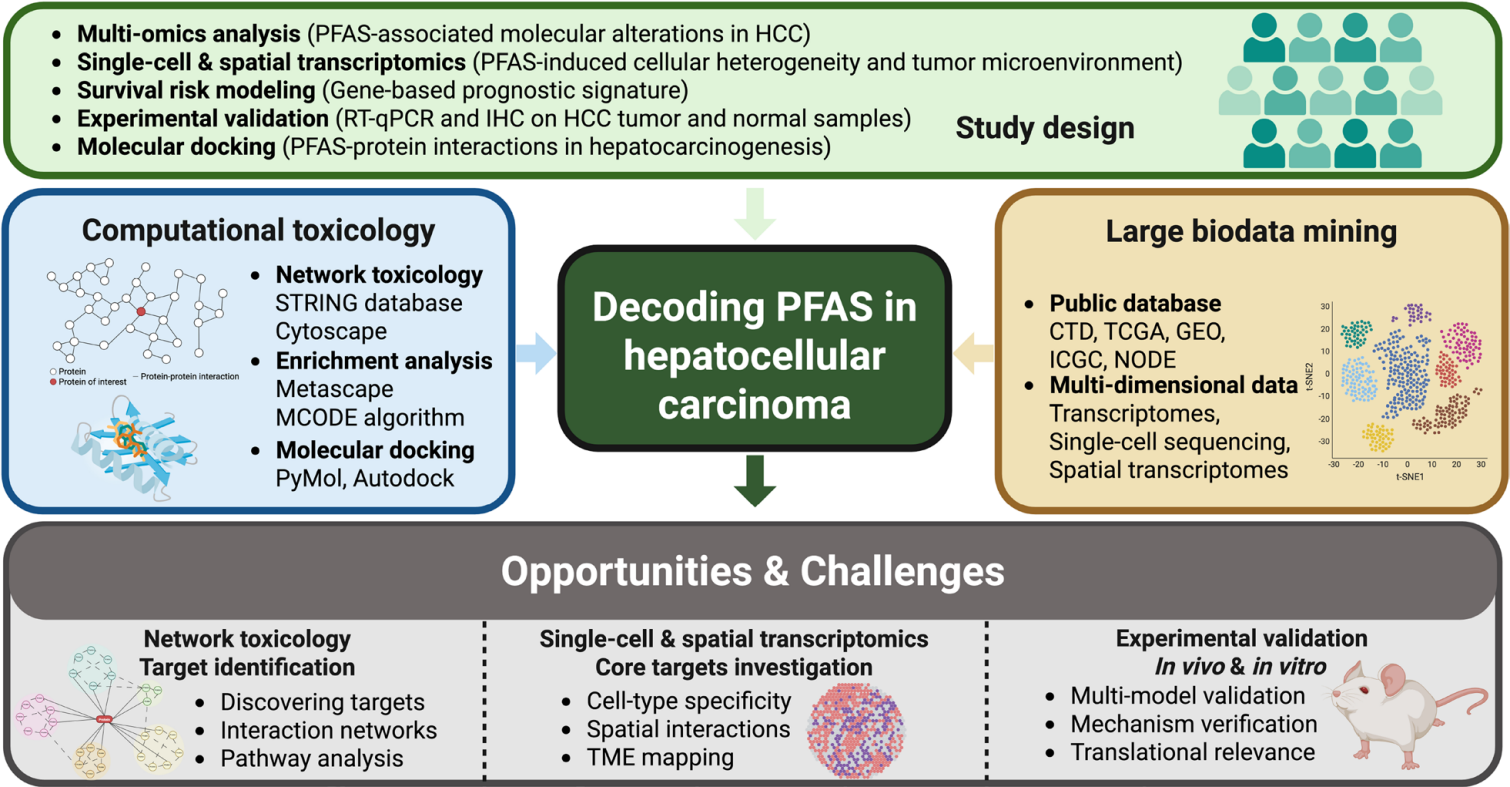
A schematic illustration of the overall workflow of this study integrating multi-omics data and computational toxicology to investigate the impact of PFAS (PFOA and PFOS) exposure on HCC. The approach included target identification using toxicogenomic databases, network toxicology analysis, multi-cohort transcriptomic data analysis, construction of a survival risk model (PFASRHSig), validation with scRNA-seq and spatial transcriptomics, and experimental confirmation via RT-qPCR, IHC, and molecular docking simulations

To address these gaps, this study employs a multi-omics approach integrating network toxicology, machine learning combinations, single-cell sequencing, spatial transcriptomics, experimental validation, and molecular docking to systematically investigate the mechanisms through which PFAS exposure contributes to hepatocarcinogenesis. Specifically, we hypothesize that perfluorooctanoic acid (PFOA) and perfluorooctane sulfonate (PFOS), the two most prevalent PFAS compounds [9], may promote HCC development by interacting with specific molecular targets and signaling pathways involved in tumorigenesis. By identifying key PFAS-related HCC targets and constructing a robust PFAS-related HCC prognostic model, this study aims to provide novel insights into PFAS-induced liver carcinogenesis and potential therapeutic targets (Fig. 1).

## Materials and methods

### Datasets selection

This study employs a multi-omics and computational toxicology approach to investigate the molecular mechanisms through which PFAS contribute to HCC progression. Bulk RNA-seq, single-cell RNA-seq (scRNA-seq), and spatial transcriptomics datasets from the TCGA, ICGC, GEO, and NODE data portal were analyzed to identify differentially expressed genes (DEGs) and their involvement in HCC. The dataset selection criteria were as follows: (i) bulk RNA-seq data should contain detailed patient clinical traits; (ii) scRNA-seq data should contain a large number of cells and various cell types; and (iii) spatial transcriptomic data should include both malignant and adjacent normal tissues. The dataset IDs are presented in the Results section.

### Compilation of targets of PFOA and PFOS

This study focused on PFOA and PFOS because of their dominant presence in environmental and biological matrices and their potential role in hepatocellular carcinogenesis. To ensure structural accuracy, we first obtained the chemical structures and canonical SMILES representations of PFOA and PFOS from the PubChem database (https://pubchem.ncbi.nlm.nih.gov). Next, we identified potential human protein targets via SwissTargetPrediction (http://www.swisstargetprediction.ch), filtering for targets specific to ‘*‘Homo sapiens’’* and retaining only those with a prediction probability greater than zero to increase reliability [19, 20]. Additionally, we queried the Comparative Toxicogenomics Database (CTD, https://ctdbase.org/) via the keywords ‘‘Perfluorooctanoic acid’’ and ‘‘Perfluorooctane sulfonic acid’’, restricting the search to human-associated targets [21]. To ensure robustness, we included only targets with at least one supporting reference in the CTD. Finally, we merged the targets from both databases and standardized their nomenclature via the UniProt database (https://www.uniprot.org) to eliminate redundancies and inconsistencies [22]. This streamlined process ensures the accuracy and reliability of the compiled target dataset, forming a solid foundation for subsequent network toxicology and molecular mechanism investigations.

### Identification of targets associated with HCC

In this study, we prioritized differentially expressed genes (DEGs) that also interact with PFAS to identify biologically relevant targets in HCC. While PFAS can interact with various proteins, not all these interactions necessarily lead to measurable changes in gene expression. Therefore, we focused on genes that were both differentially expressed in HCC and predicted PFAS targets, ensuring that our analysis highlights functionally significant genes that are not only involved in PFAS interactions but also actively contribute to HCC progression. This approach allows us to pinpoint genes with a greater likelihood of playing a role in PFAS-induced hepatocarcinogenesis rather than including all potential PFAS-interacting proteins that may not exhibit transcriptional changes. The differential gene expression analysis was based on transcriptomic data from the TCGA-LIHC (Liver Hepatocellular Carcinoma) cohort obtained from the UCSC Xena data portal (https://xena.ucsc.edu/). Gene expression profiles from tumor and adjacent normal liver tissues were analyzed via the DESeq2 R package, with a false discovery rate (FDR) < 0.05 and |log_2_FoldChange| > 1 used as thresholds to determine significantly upregulated and downregulated genes. The identified HCC-related DEGs were then compared with PFAS-associated targets compiled from SwissTargetPrediction and the CTD. A Venn diagram was constructed via an online bioinformatics tool (http://bioinformatics.psb.ugent.be/webtools/Venn/) to identify overlapping targets that were both significantly dysregulated in HCC and predicted to interact with PFOA and PFOS. These shared targets were prioritized for downstream network analysis, machine learning modeling, and functional validation, providing deeper insights into the molecular mechanisms linking PFAS exposure to hepatocarcinogenesis.

### PPI network construction and selection of top targets

To explore the interactions between common PFAS and HCC-associated targets, we constructed a protein–protein interaction (PPI) network via the STRING database (http://string-db.org). The analysis was restricted to *"Homo sapiens"*, with a minimum confidence score of 0.40 to ensure biologically relevant interactions [23]. The resulting network was visualized in Cytoscape (version 3.10.2), where key topological properties, including degree centrality, closeness centrality, and betweenness centrality, were assessed to identify crucial hub proteins [24]. To refine the target selection, we identified 83 key targets with a degree ≥ median degree value for further machine learning analysis. Additionally, to determine the top 20 hub targets with the most significant network influence, we employed the CytoHubba plugin in Cytoscape, applying four different ranking algorithms, which included Maximum Neighborhood Component (MNC), Degree, Edge Percolated Component (EPC), and Closeness [25]. These high-confidence targets were prioritized for downstream analysis, including survival modeling, to elucidate their potential role in PFAS-mediated hepatocarcinogenesis.

### Gene ontology and pathway enrichment analyses

To gain deeper insights into the biological functions and pathways associated with common PFAS and HCC-associated targets, we conducted gene ontology (GO) and pathway enrichment analyses via Metascape (http://metascape.org/) [26]. The analysis included functional annotation of the 174 identified common targets, covering biological processes, molecular functions, and cellular components. We applied a minimum overlap of 3 genes, a *P*-value cutoff of 0.05, and a minimum enrichment score of 1.5 to ensure statistical significance. Additionally, we performed Kyoto Encyclopedia of Genes and Genomes (KEGG) pathway enrichment to identify key molecular pathways affected by PFAS exposure in HCC. PPI networks were further analyzed via the MCODE algorithm to identify highly interconnected clusters of proteins, revealing potential mechanistic modules. The enrichment results were visualized via ggplot2 in R and Cytoscape, highlighting the core biological processes and signaling pathways underlying PFAS-induced hepatocarcinogenesis.

### Integrative machine learning approach

To establish a reliable survival risk framework associated with FPAS, namely, the PFAS-related HCC signature (PFASRHSig), we integrated 10 machine learning algorithms and 101 algorithm combinations, including Lasso, Ridge, Enet, StepCox, survivalSVM, CoxBoost, SuperPC, plsRcox, RSF, and GBM. Initially, univariate Cox regression was employed to identify prognostic genes in the TCGA-LIHC cohort. We subsequently applied the 101 algorithm combinations to these genes to construct predictive models, which were evaluated via leave-one-out cross-validation (LOOCV) [27] within the TCGA-LIHC cohort. The models were then tested on five external validation datasets (CHCC, LICA, LIRI, GSE14520 and GSE54236). For each model, Harrell’s concordance index (C-index) was calculated across all datasets, and the model with the highest average C-index was selected as the optimal model. The R package"Mine1"was utilized to implement the LOOCV framework for machine learning [28].

### Model assessment

To assess the reliability and predictive power of the PFASRHSig survival risk model, we applied a detailed evaluation strategy, incorporating Kaplan–Meier (KM) survival analysis and ROC-AUC analysis [29]. In the KM analysis, patients were categorized into high-risk and low-risk groups on the basis of the median values from six distinct cohorts. Statistical significance was determined via the Log-Rank test. Furthermore, we conducted ROC-AUC analysis to evaluate the diagnostic accuracy of the model, calculating the AUC value as an indicator of its performance. This combined analysis underscores the model’s strong ability to predict patient prognosis.

### Single-cell analysis

For scRNA-seq analysis, we applied strict quality control criteria to ensure data reliability. The cells were retained if they contained fewer than 20% mitochondrial gene content, expressed more than 200 genes, and had between 200 and 6000 detected genes, which appeared in at least three cells. A total of 71,915 high-quality cells were selected for downstream analysis. To correct batch effects and improve clustering accuracy, we utilized the Harmony algorithm for data integration [30]. The data were normalized via log-normalization, and the FindVariableFeatures function was used to identify the top 2000 highly variable genes. Principal Component Analysis (PCA) was performed for dimensionality reduction, followed by soft k-means clustering via the Harmony package. The cells were then grouped into distinct clusters via the FindClusters function with a resolution of 0.4. Cell type annotation was conducted on the basis of canonical marker genes, differential expression patterns, and known cellular profiles [31]. The expression distribution of key PFAS-related target genes across these cell types was further analyzed to uncover potential cellular mechanisms linking PFAS exposure to HCC progression.

### Spatial transcriptomics

For spatial transcriptomics analysis, we utilized the Seurat R package to preprocess, normalize, and scale the raw spatial transcriptomics data. Tumor cell identification, ecosystem characterization, and cell type deconvolution were performed via SpaCET (v1.0.0) [32]. SpaCET determines cancer cell fractions via a gene pattern dictionary that captures copy number alterations and expression variations across different tumor types. This approach outperforms the conventional inferCNV-based method in both predictive accuracy and computational efficiency. For non-malignant cells, SpaCET employs a hierarchical two-tier model to break down their composition. At the first tier, the proportions of primary cell lineages are estimated, whereas at the second tier, the sublineage fractions are inferred on the basis of their corresponding major lineage fractions. These estimations utilize reference profiles derived from scRNA-seq datasets spanning multiple cancer types [33]. This approach enables precise mapping of cellular heterogeneity within the HCC tumor microenvironment, facilitating the investigation of PFAS-associated molecular alterations in distinct spatial contexts.

### RT-qPCR

Paired tumor and adjacent normal tissues were collected from 6 patients with histologically confirmed HCC at the First Affiliated Hospital of Wenzhou Medical University (Approval No. KY2022-R141). The tissue samples were immediately snap-frozen in liquid nitrogen and stored at − 80 °C until further processing. Total RNA was extracted via TRIzol reagent (Invitrogen) in accordance with the manufacturer’s instructions. Reverse transcription was performed using Superscript II reverse transcriptase (Invitrogen) and gene-specific primers. Real-time quantitative PCR (RT-qPCR) was conducted via the ABI Prism 7300 Sequence Detection System (Applied Biosystems) to assess the expression of ESR1, APOA1, IGF1, PPARGC1A, SERPINE1, PON1, and the reference gene β-actin. Relative transcript levels were normalized to β-actin expression via the relative standard curve method, following the manufacturer’s technical guidelines (Applied Biosystems). All data analysis adhered to the MIQE guidelines [34], ensuring experimental transparency and reproducibility. The detailed primer sequences are provided in Supplementary Table 6. The significant differences were analyzed by one-way ANOVA and t-test via GraphPad Prism 10.1.2 (San Diego, CA, USA). *P* < 0.05 was considered to indicate a significant difference.

### Immunohistochemical (IHC) staining

IHC images of both tumor and normal tissues were obtained from the Human Protein Atlas (https://www.proteinatlas.org/). However, IHC data for PPARGC1A were not available in the database and were therefore not included in the analysis.

### Molecular docking

To validate the interactions between PFAS compounds (PFOA and PFOS) and the core target proteins, molecular docking simulations were performed via a semi-flexible docking strategy [35]. The crystal structures of the core proteins were obtained from the AlphaFold Protein Structure Database (AlphaFold DB, https://alphafold.ebi.ac.uk) [36]. Protein structures were preprocessed via PyMOL (version 1.8.6) to remove water molecules and ligands, followed by optimization via the AutoDock and AutoDock Vina tools (version 1.5.7) [37]. A grid box was defined for each target protein to specify the active site for docking. Ten docking runs were conducted for each protein–ligand pair with a population size of 150, a maximum of 2,500,000 evaluations, a mutation rate of 0.02, and a crossover rate of 0.8 [38]. The docking results were analyzed on the basis of binding energies and interaction sites, and the interactions were visualized via PyMOL and Discovery Studio 2024, with a focus on key bonding interactions such as hydrogen bonds and hydrophobic contacts. This process helps assess the binding affinities and potential molecular mechanisms of PFAS interactions with the identified core targets.

## Results

### Selection of bulk RNA-seq, scRNA-seq, and spatial transcriptomic datasets

Bulk RNA-seq datasets with detailed patient clinical traits were selected. We retrieved six publicly available datasets, namely, the TCGA-LIHC cohort (n = 355) from the UCSC Xena data portal (https://xena.ucsc.edu/), the LIRI (n = 202) and LICA (n = 152) cohorts from the ICGC data portal (https://dcc.icgc.org/projects), the CHCC cohort (n = 159) from the NODE database (https://www.biosino.org/node), and the GSE14520 (n = 221) and GSE54235 (n = 78) cohorts from the GEO data portal (https://www.ncbi.nlm.nih.gov/geo/). Regarding the scRNA-seq data, the GSE149614 cohort (n = 10) was selected because of its large number of cells and various cell types included. We then selected patial transcriptomic sequencing data containing both malignant and adjacent normal tissues, which were downloaded from https://ngdc.cncb.ac.cn/gsa-human/browse/HRA000437 [39].

### PPI network of potential targets and identification of top targets

**Fig. 2:**
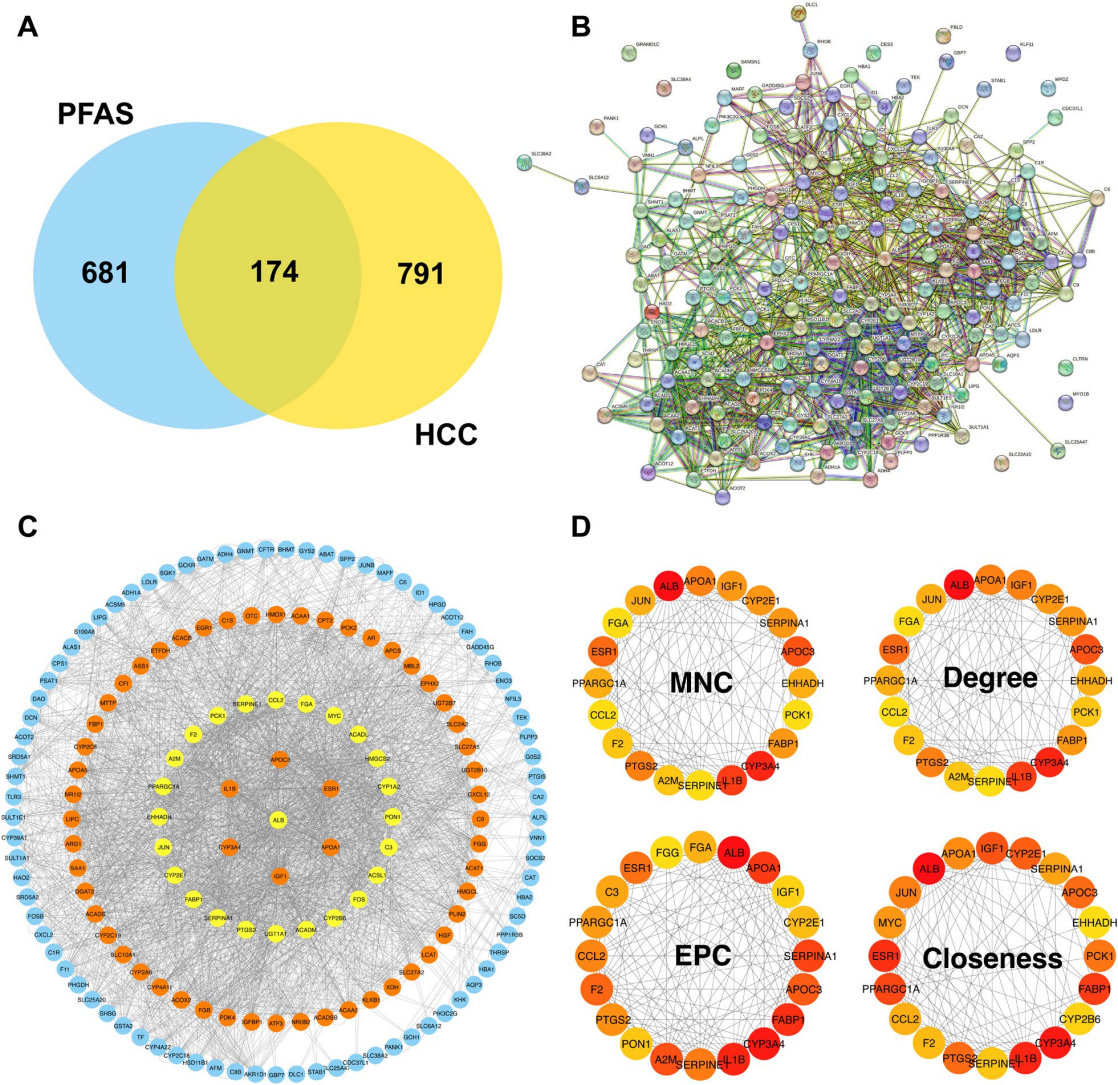
Identification of common PFAS and HCC-associated targets and PPI network analysis. **A** Venn diagram showing the overlap between 855 PFAS-associated targets (derived from SwissTargetPrediction and CTD databases) and 965 differentially expressed genes (DEGs) in HCC (obtained from TCGA-LIHC, n = 355, tumor vs. adjacent normal liver tissues). Differential expression was determined using DESeq2 with |log2 FoldChange| > 1 and FDR < 0.05. **B** PPI network of the 174 overlapping targets constructed using the STRING database (confidence score > 0.40; organism: *Homo sapiens*). **C** Visualization of the STRING-based PPI network in Cytoscape (version 3.10.2), showing interaction complexity and node connectivity. **D** Top 20 hub genes identified using the CytoHubba plugin in Cytoscape based on four ranking algorithms: MNC, Degree, EPC, and Closeness

A total of 855 targets for PFOA and PFOS were identified from the CTD and SwissTargetPrediction databases (Supplementary Table 1). Additionally, we identified 965 targets highly relevant to HCC via data from the TCGA-LIHC cohort (Supplementary Table 2). Among them, 174 common targets were found (Fig. 2A). To further analyze the interactions among these targets, we constructed a PPI network via the STRING database, which comprised 174 nodes and 1341 edges, with an average node degree of 15.4 (Fig. 2B). These 174 targets and their associations were subsequently imported into Cytoscape for further visualization (Fig. 2C, Supplementary Table 3). A total of 83 targets with degrees ≥ the median value (degree = 30) of degree were further identified. The top 20 targets analyzed via the CytoHubba plug-in applying four different algorithms (MNC, Degree, EPC, and Closeness) were additionally identified, and among these, 17 common top targets were identified (Fig. 2D).

### Enrichment analysis of 174 common PFAS-related mechanisms in HCC

**Fig. 3:**
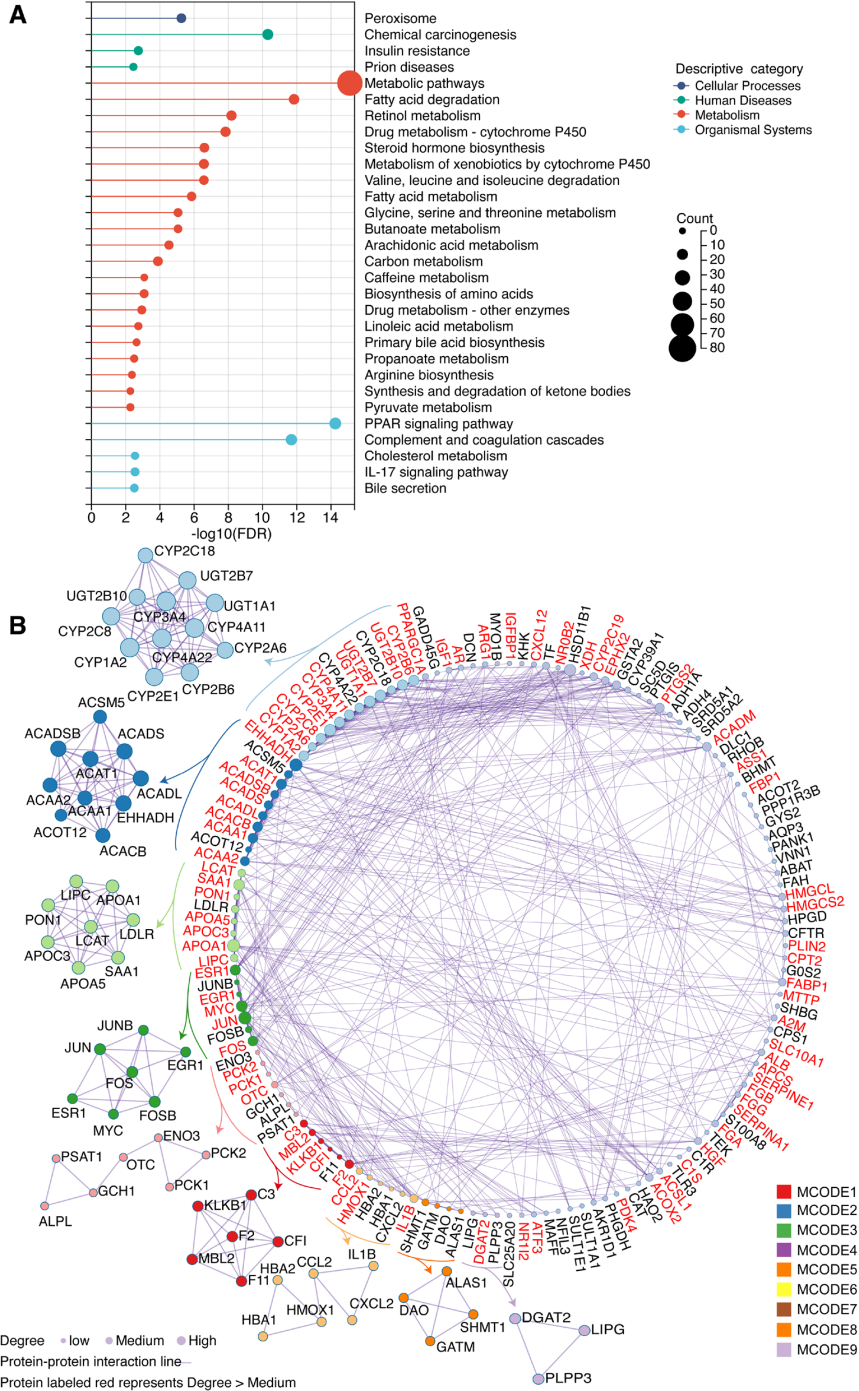
Functional enrichment and PPI clustering analysis of common PFAS and HCC-associated targets. **A** KEGG pathway enrichment of the 174 overlapping PFAS-HCC targets using Metascape. Pathways are grouped into metabolism, immune regulation, and cancer-related processes. Enrichment significance was evaluated using the hypergeometric test, with Benjamini–Hochberg correction (adjusted *P* < 0.05). **B** PPI network analysis using the MCODE algorithm identified nine functional modules enriched in metabolic processes, drug metabolism, and tumor progression. Visualization created using Cytoscape (version 3.10.2)

To investigate the potential biological functions and pathways associated with PFAS-related targets in HCC, we performed functional enrichment analysis via Metascape. A total of 174 common targets were analyzed for GO biological processes, molecular functions, and cellular components, alongside KEGG pathway enrichment. The results revealed that these targets were enriched in pathways such as the carboxylic acid metabolic process, nuclear receptors meta pathway, steroid metabolic process, liver carcinoma, and response to xenobiotic stimulus (Table 1). The KEGG results revealed significant enrichment in pathways related to metabolism (fatty acid metabolism, steroid metabolism), oncogenic processes (chemical carcinogenesis, PPAR signaling), and immune response (complement and coagulation cascades, IL-17 signaling) (Fig. 3A, Supplementary Table 4). Additionally, PPI network analysis revealed nine functional modules via the MCODE algorithm, highlighting tightly connected clusters involved in lipid regulation, drug metabolism, and tumor progression (Fig. 3B, Supplementary Table 5). These findings provide crucial insights into the molecular mechanisms by which PFAS exposure contributes to HCC development and progression.

**Table 1:** Top 20 terms in the enrichment analysis ranked by *P*-value

Category	Term	Description	− Log10P	nCount
GO Biological Processes	GO:0019752	Carboxylic acid metabolic process	55.35	85
WikiPathways	WP2882	Nuclear receptors meta pathway	42.93	41
GO Biological Processes	GO:0008202	Steroid metabolic process	37.16	58
GeDiPNet	GDP04174	Liver carcinoma	31.92	41
GO Biological Processes	GO:0009410	Response to xenobiotic stimulus	27.73	72
GO Biological Processes	GO:0044282	Small molecule catabolic process	26.78	54
WikiPathways	WP3942	PPAR signaling	22.90	22
GeDiPNet	GDP03928	Kidney Failure	22.79	25
GeDiPNet	GDP02569	Fatty Liver	21.12	31
GO Biological Processes	GO:0031667	Response to nutrient levels	20.91	30
KEGG Pathway	hsa04610	Complement and coagulation cascades	20.80	40
GeDiPNet	GDP01265	Cholestasis	19.16	20
WikiPathways	WP15	Selenium micronutrient network	17.61	38
GeDiPNet	GDP04906	Myocardial Infarction	16.96	35
WikiPathways	WP4545	Oxysterols derived from cholesterol	15.94	21
WikiPathways	WP3925	Amino acid metabolism	15.67	38
GeDiPNet	GDP04907	Myocardial Ischemia	15.50	38
GO biological processes	GO:0010876	Lipid localization	15.04	30
GO biological processes	GO:0010038	Response to metal ion	14.94	23
GO biological processes	GO:0062012	Regulation of small molecule metabolic process	14.84	25

### Construction and assessment of the PFASRHSig survival risk framework

**Fig. 4:**
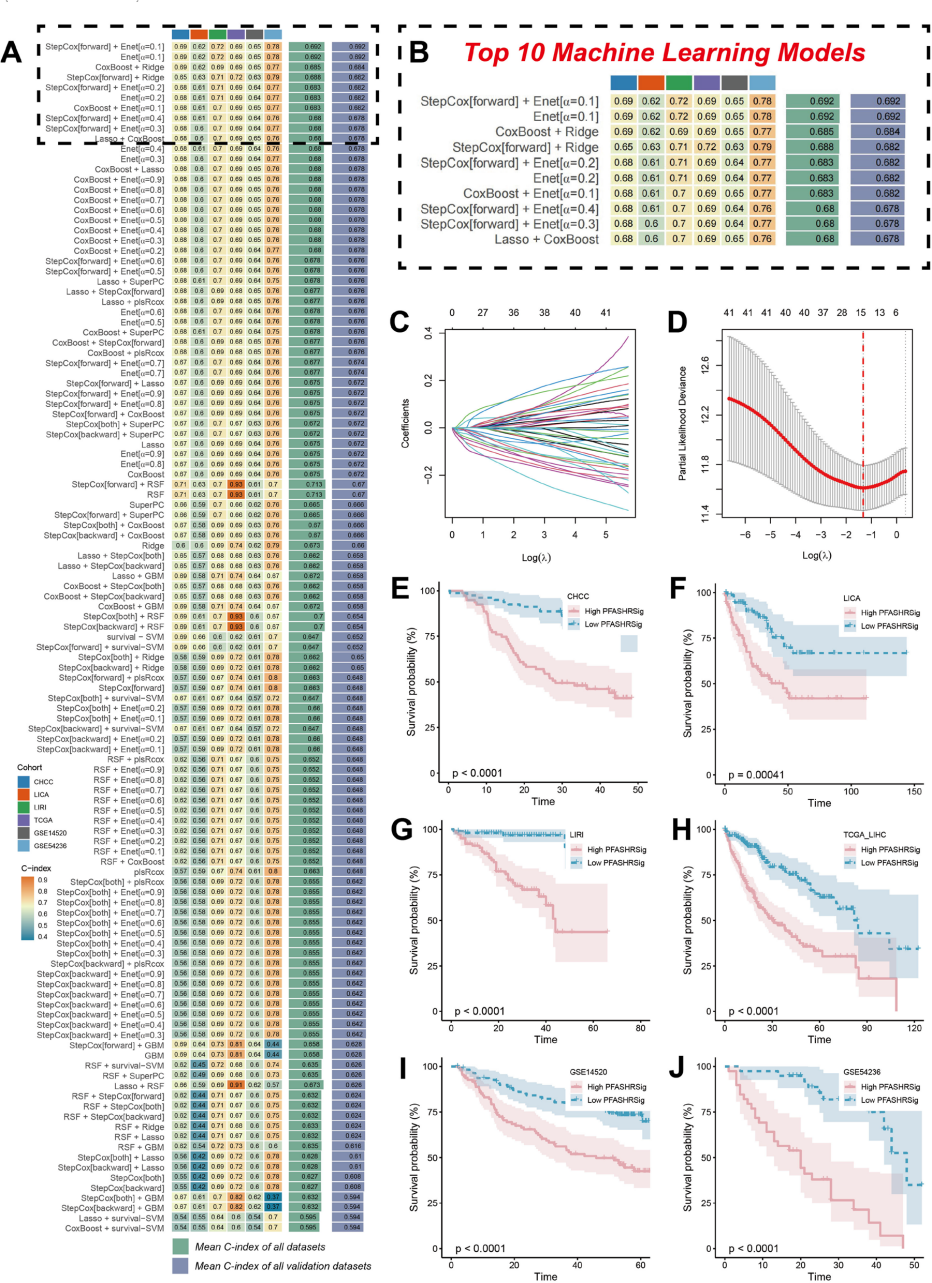
Construction of the PFASRHSig survival risk model using multi-cohort transcriptomic data and machine learning. **A** Heatmap showing concordance index (C-index) of 101 machine learning models across six datasets: TCGA-LIHC (n = 355), CHCC (n = 159), LICA (n = 152), LIRI (n = 202), GSE14520 (n = 221), and GSE54236 (n = 78). **B** Top 10 predictive models by mean C-index. **C** Elastic net regression coefficient profiles across different values of log(λ). **D** Optimal λ selection using cross-validation with minimum partial likelihood deviance. (E-J) Kaplan–Meier survival curves comparing high- and low-risk groups (median cutoff) in six cohorts. Statistical significance was determined using the log-rank test

Using the expression data of 84 targets with a degree ≥ 30, we performed univariate Cox regression, which revealed 41 genes with prognostic value. These identified genes were then integrated into a machine learning framework to develop a unified risk model, which was validated across four distinct datasets. For the TCGA-LIHC cohort, we constructed 101 predictive models through the LOOCV approach, and the C-index for each model was computed across all the validation datasets (Fig. 4A).

Among the models with comparable C-index values, those incorporating fewer hub genes were preferred. The optimal model combined stepwise Cox regression (forward direction) with Enet (α = 0.1) regression, achieving the highest average C-index (0.692) while retaining the fewest genes (Fig. 4B). The stepwise Cox regression (forward direction) retained all 41 prognostic genes, whereas the Enet regression determined the optimal λ by minimizing partial likelihood deviance (Fig. 4C). Ultimately, 14 genes with nonzero Enet coefficients were selected (Fig. 4D). The resulting risk model was defined as follows: Risk Score = exp(−0.0136531033 × ESR1 − 0.0057401447 × APOA1 − 0.0127939803 × IGF1 − 0.0601279664 × PPARGC1A + 0.0273195307 × SERPINE1 + 0.0476269799 × HMOX1 − 0.0001355368 × APCS − 0.0629751524 × ACADS − 0.0080130999 × SLC10 A1 − 0.0245156728 × SLC2 A2 − 0.0469738128 × ACAT1 − 0.0089196823 × C1S − 0.0848350866 × LCAT).

**Fig. 5:**
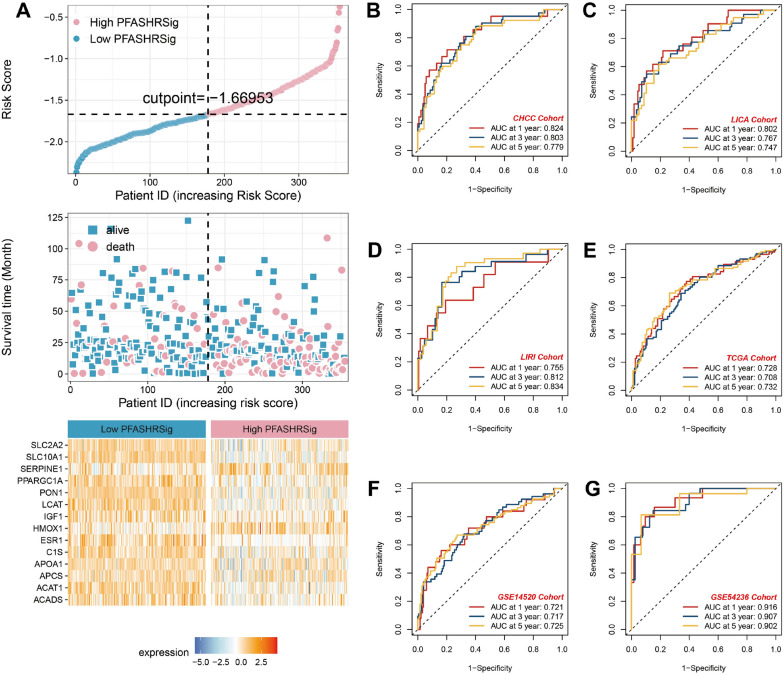
Assessment of the predictive performance of PFASRHSig. **A** Risk score distribution in the TCGA-LIHC cohort. The cutpoint is set at − 1.66953, the median risk score. The heatmap presents the varied expression levels of model genes across high and low PFASRHSig group. (B-G) Time-dependent ROC curves assessing 1-, 3-, and 5-year survival prediction across six cohorts: CHCC (n = 159)-AUC: 0.824, 0.803, 0.779; LICA (n = 152)-AUC: 0.802, 0.767, 0.747; LIRI (n = 202)-AUC: 0.755, 0.812, 0.834; TCGA-LIHC (n = 355)-AUC: 0.728, 0.708, 0.732; GSE14520 (n = 221)-AUC: 0.721, 0.717, 0.725; GSE54236 (n = 78)-AUC: 0.916, 0.907, 0.902

As illustrated in Fig. 4E–J, patients classified into the high-risk group exhibited significantly poorer OS than those in the low-risk group across the TCGA-LIHC training dataset and five validation datasets (all *P* < 0.001) (Fig. 5A). The model’s discriminative ability was assessed via ROC-AUC analysis, yielding AUC values for 1-, 3-, and 5 year survival rates of 0.728, 0.708, and 0.732, respectively, in the TCGA-LIHC cohort; 0.824, 0.803, and 0.779, respectively, in the CHCC cohort; 0.802, 0.767, and 0.747, respectively, in the LICA cohort; 0.755, 0.812, and 0.834, respectively, in the LIRI cohort; 0.721, 0.717, and 0.725, respectively, in the GSE14520 cohort; and 0.916, 0.907, and 0.902, respectively, in the GSE54236 dataset (Fig. 5B–G).

### Cellular landscape of HCC mapped through scRNA-seq data

**Fig. 6:**
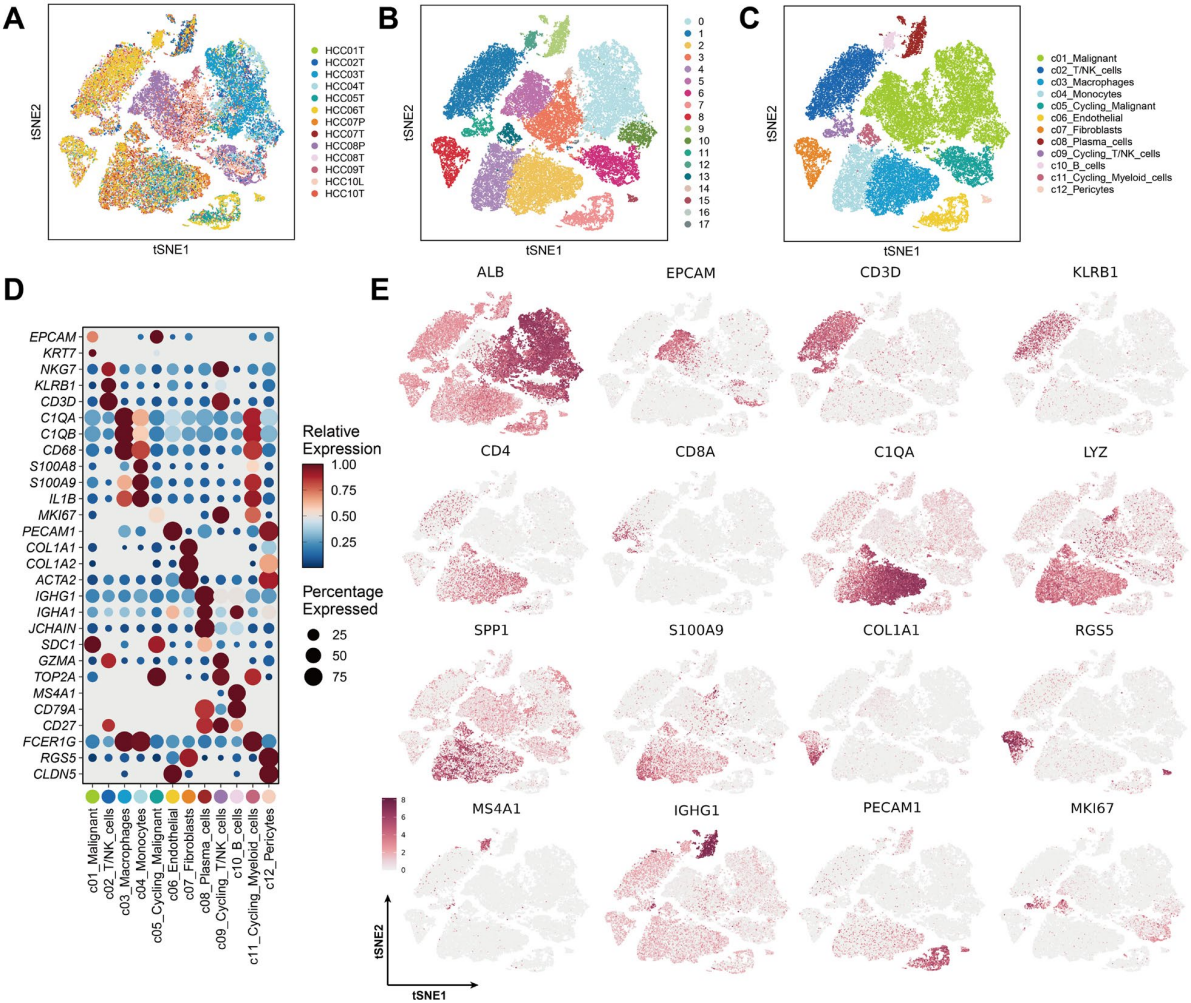
Cellular landscape of HCC from scRNA-seq analysis. (A) UMAP plot illustrating batch-corrected integration of 13 scRNA-seq samples (GSE149614), comprising 10 HCC patients, using the Harmony algorithm. **B** Clustering of 71,915 high-quality cells into 17 distinct clusters using the Seurat R package (resolution = 0.4). **C** Annotation of cell types based on canonical markers: T/NK cells, B cells, plasma cells, malignant cells, macrophages, monocytes, fibroblasts, endothelial cells, pericytes, and cycling cells. **D** Dot plot showing expression patterns of classical marker genes used to annotate cell types in different clusters. **E** UMAP plot visualizing expression patterns of key marker genes

After the Harmony algorithm was applied, the cellular distribution within each sample remained largely consistent, indicating the absence of significant batch effects among the samples, making them suitable for downstream analysis (Fig. 6A). A total of 17 distinct cell clusters were identified using a resolution parameter of 0.4 (Fig. 6B). We reanalyzed 71,915 scRNA-seq cells spanning 13 samples, including primary tumors, lymph node tissues and portal vein tumor thrombi (PVTTs). On the basis of classical marker genes, we successfully classified various cell populations, including T/NK cells, B cells, plasma cells, malignant cells, monocytes, macrophages, fibroblasts, pericytes, endothelial cells and cycling cells (Fig. 6C). The marker genes corresponding to each cell type displayed distinct expression patterns, reinforcing the accuracy of our cell annotation (Fig. 6D, 6E).

### Expression profiles of the core targets within the HCC ecosystem

**Fig. 7:**
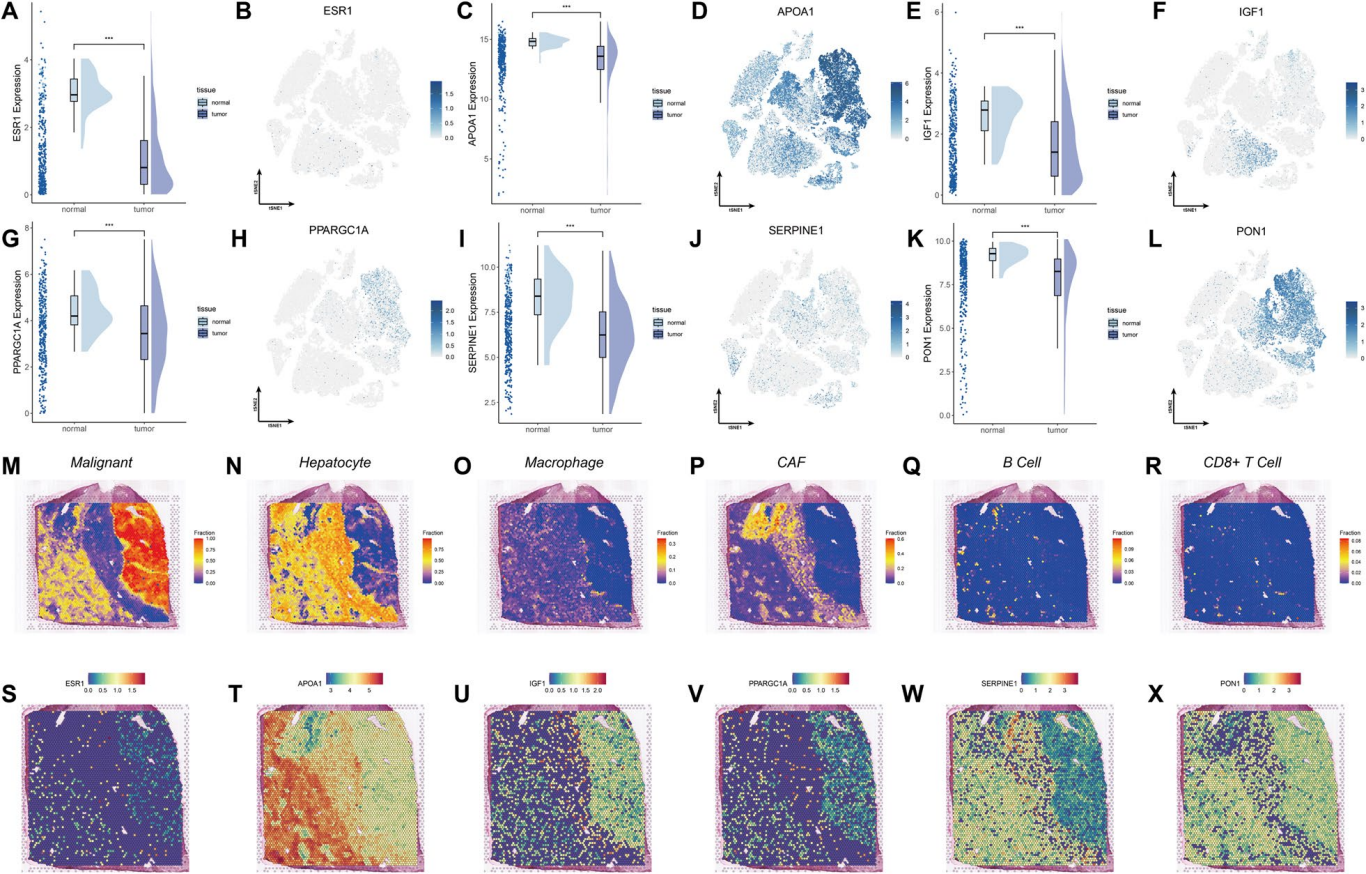
Expression and spatial distribution of six core PFAS-HCC target genes. **A**–**L** Box plots and UMAP visualizations showing the differential expression of ESR1, APOA1, IGF1, PPARGC1A, SERPINE1, and PON1 in tumor vs. adjacent normal tissues. Statistical significance assessed via unpaired t-test. (M-R) Cell type annotation maps from spatial transcriptomics, showing distribution of malignant cells, hepatocytes, macrophages, CAFs, B cells, and CD8 + T cells. **S**–**X** Spatial gene expression maps of ESR1, APOA1, IGF1, PPARGC1A, SERPINE1, and PON1. *P < 0.05; **P < 0.01; ***P < 0.001

The 41 prognostic gene targets identified from the PFASRHSig survival risk framework overlapped with the 17 common top targets identified via the CytoHubba plugin, resulting in the identification of six core targets for further analysis. All six core targets presented significantly higher expression levels in normal tissues than in tumor tissues (Fig. 7A, 7 C, 7E, 7G, 7I, and 7 K). Notably, APOA1 was abundant in both tumor and normal tissues, highlighting its crucial roles in cellular biology (Fig. 7D). Single-cell landscape analysis revealed low ESR1 expression across all cell types within the HCC ecosystem (Fig. 7B), whereas APOA1, PPARGC1A, SERPINE1, and PON1 were predominantly expressed in malignant cells (Fig. 7D, 7H, 7 J, and 7L). Among all cell types, macrophages presented the highest concentration of IGF1 (Fig. 7F).

We further investigated the expression profiles of these six core genes via spatial transcriptomics. Various cell types within the HCC microenvironment, including malignant cells, hepatocytes, and several types of stromal cells, were annotated (Fig. 7M–R). ESR1 was slightly more highly expressed in normal hepatocytes than in malignant cells, with relatively lower expression levels observed in stromal cells (Fig. 7S). In contrast, APOA1 was significantly more highly expressed in normal and stromal tissues than in malignant tissues (Fig. 7T). IGF1, PPARGC1A, SERPINE1, and PON1 exhibited similar distribution patterns (Fig. 7U–X).

### Validation of target gene expression by RT-qPCR and IHC

**Fig. 8:**
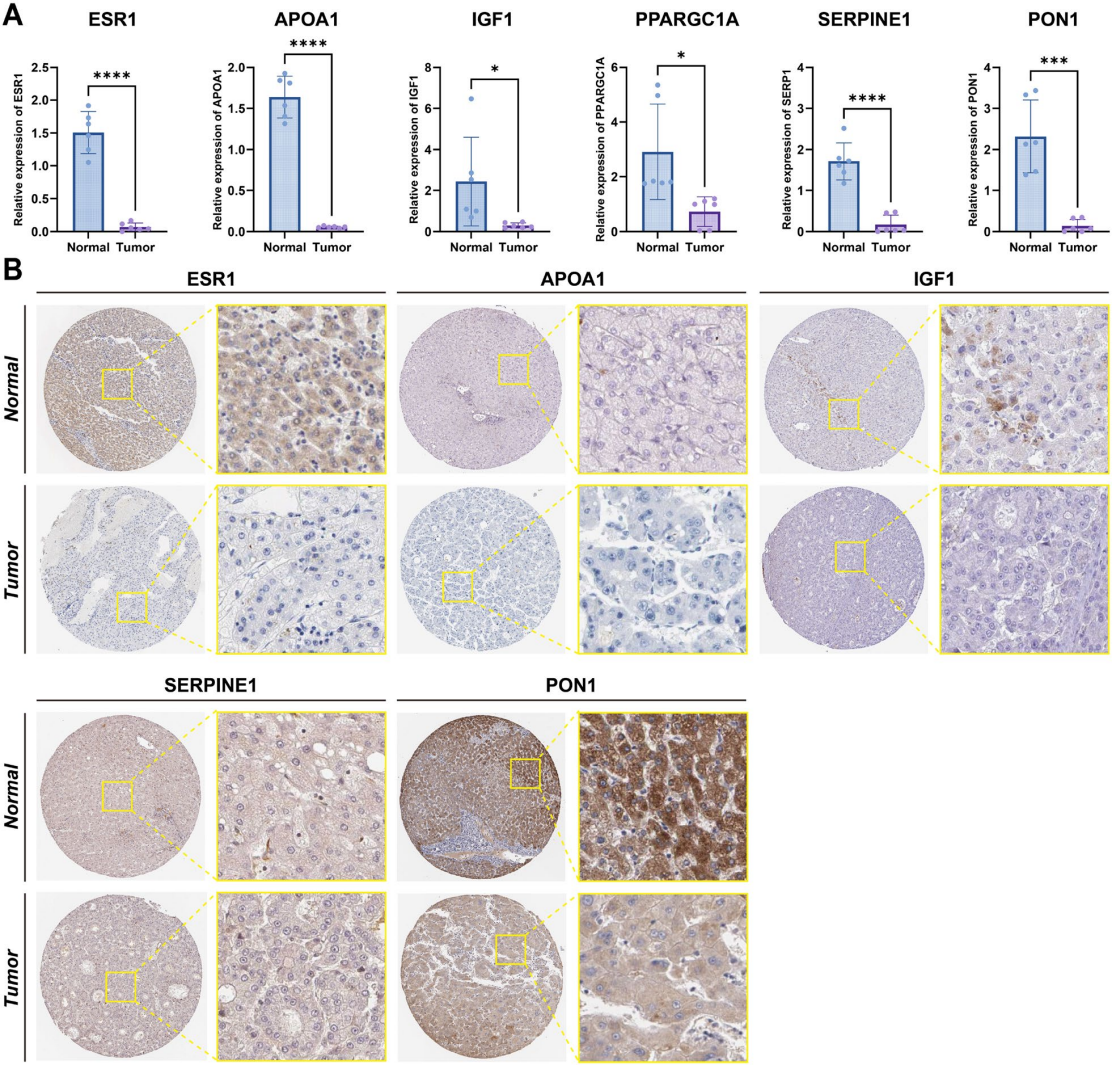
Experimental validation of gene expression by RT-qPCR and IHC. **A** RT-qPCR quantification of gene expression (ESR1, APOA1, IGF1, PPARGC1A, SERPINE1, and PON1) in paired tumor and adjacent normal tissues from six HCC patients. Data presented as mean ± SD; significance determined by paired t-test (*P* < 0.05). **B** IHC images (sourced from Human Protein Atlas) showing protein expression patterns. Strong staining in normal tissues and reduced or absent staining in tumor tissues for ESR1, APOA1, IGF1, and PON1. SERPINE1 showed comparable staining in both tissue types. PPARGC1A IHC data not available. *P < 0.05; **P < 0.01; ***P < 0.001; ****P < 0.0001

To validate the expression patterns of the six core genes identified in silico, we performed RT-qPCR and IHC staining on tumor and normal tissue samples. The RT-qPCR analysis revealed significantly decreased expression of ESR1, APOA1, IGF1, PPARGC1A, SERPINE1 and PON1 in tumor tissues compared with adjacent normal tissues (*P* < 0.05) (Fig. 8A). Consistent with these findings, the IHC staining results of the six core targets demonstrated similar trends at the protein level (Fig. 8B). Strong immunoreactivity of ESR1, APOA1, IGF1, and PON1 was observed in normal tissues, whereas weak or no staining was observed in tumor tissues. SERPINE1 showed similar staining intensities in tumor and normal tissues.

### Molecular docking of PFOA and PFOS with 6 core target proteins of HCC

**Fig. 9:**
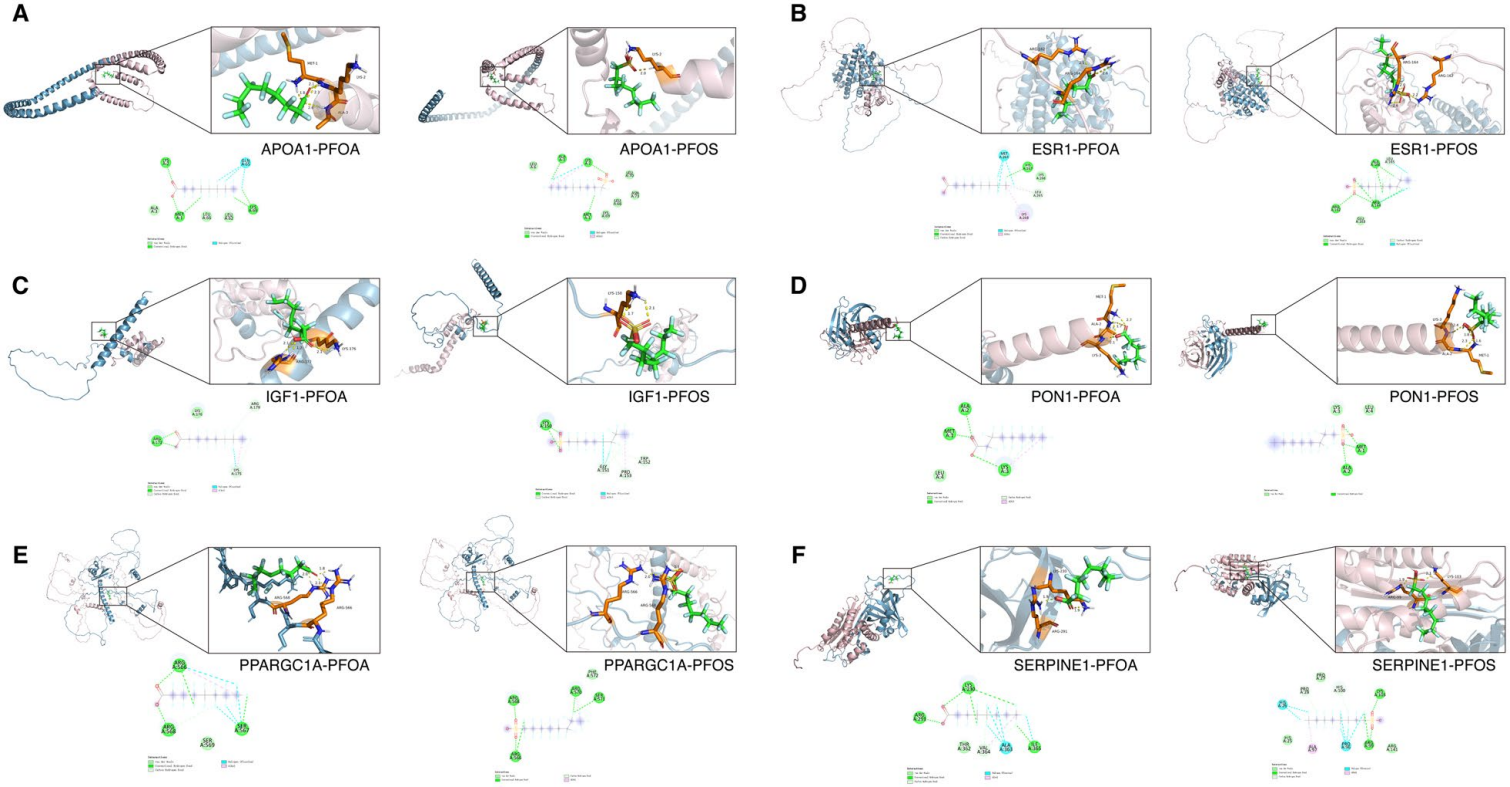
Molecular docking results of PFAS with six core HCC target proteins. **A**–**F** Visualization of docking interactions between PFOA and PFOS with APOA1, ESR1, IGF1, PON1, PPARGC1A, and SERPINE1. Docking was performed using AutoDock Vina. Binding energy values ranged from − 5.3 to − 7.6 kcal/mol, indicating spontaneous binding. Interaction types included van der Waals forces, hydrogen bonds, halogen interactions, and alkyl contacts. Detailed interactions are listed in Table 2

We performed molecular docking analysis to explore the interactions between PFOA, PFOS and six core target proteins: APOA1, ESR1, IGF1, PPARGC1A, SERPINE1 and PON1. The docking results, obtained via AutoDock and AutoDock Vina Tools, revealed strong affinities between PFOA, PFOS and six target proteins (Table 2). These findings suggest that binding is spontaneous and highlight the critical role of these proteins in the mechanism of PFOA and PFOS in HCC. The interactions between PFOA, PFOS and the six core target proteins were visualized and are depicted in Fig. 9.

**Table 2:** Molecular docking results of PFOA and PFOS with 6 core target proteins

Ligand	Receptor	Binding energy (kcal/mol)	Key interactions
PFOA	APOA1	− 5.3	van der Waals, Conventional Hydrogen Bond, Halogen (Fluorine)
	ESR1	− 7.2	van der Waals, Conventional Hydrogen Bond, Halogen (Fluorine), Carbon Hydrogen Bond, Alkyl
	IGF1	− 6.7	van der Waals, Conventional Hydrogen Bond, Halogen (Fluorine), Carbon Hydrogen Bond, Alkyl
	SERPINE1	− 6.3	van der Waals, Halogen (Fluorine), Conventional Hydrogen Bond, Alkyl, Carbon Hydrogen Bond
	PPARGCA1	− 6.3	van der Waals, Halogen (Fluorine), Conventional Hydrogen Bond, Alkyl, Carbon Hydrogen Bond
	PON1	− 6.7	van der Waals, Carbon Hydrogen Bond, Conventional Hydrogen Bond, Alkyl
PFOS	APOA1	− 5.5	van der Waals, Conventional Hydrogen Bond, Halogen (Fluorine), Alkyl, Carbon Hydrogen Bond
	ESR1	− 7.6	van der Waals, Conventional Hydrogen Bond, Halogen (Fluorine)
	IGF1	− 6.7	van der Waals, Conventional Hydrogen Bond, Halogen (Fluorine), Alkyl
	SERPINE1	− 6.3	van der Waals, Halogen (Fluorine), Conventional Hydrogen Bond, Alkyl, Carbon Hydrogen Bond
	PPARGCA1	− 6.4	van der Waals, Carbon Hydrogen Bond, Conventional Hydrogen Bond, Alkyl
	PON1	− 6.8	van der Waals, Conventional Hydrogen Bond

## Discussion

PFOA and PFOS are persistent organic pollutants widely used in industrial applications such as water-responsive materials and firefighting foams. Owing to their resistance to degradation, they have become common environmental contaminants, raising concerns about their potential health impacts. Prior studies have linked PFAS exposure to increased risks of liver diseases, including hepatocellular carcinoma (HCC). For example, epidemiological analyses of NHANES data suggested that high PFOS levels may increase the risk of HCC [16]. Other studies have revealed correlations between PFAS concentrations and altered liver biomarkers such as ALP, AST, and AFP in liver cancer patients [40]. In vitro experiments have also shown that PFAS may promote the proliferation and invasiveness of HCC cells [41].

While existing research has shed light on the potential hepatotoxic effects of PFAS, especially in relation to HCC, several important gaps remain. First, the majority of studies have focused primarily on epidemiological associations, with few in-depth investigations into the molecular targets and mechanisms by which PFAS contribute to HCC initiation and progression. Second, some experimental studies have evaluated the impact of PFAS on HCC cell lines by assessing changes in specific proteins, which may overlook broader biological processes and interactions, where high-throughput target screening approaches could help capture more comprehensive molecular alterations. Third, the prognostic relevance of PFAS-related molecular targets in HCC patients remains unclear, limiting their potential utility in clinical risk stratification. Finally, given the complex nature of the tumor microenvironment (TME), PFAS may also affect non-malignant cell populations, which has rarely been explored. These gaps highlight the need for a comprehensive and multi-dimensional approach.

Our study proposes a comprehensive and integrative framework to elucidate the molecular mechanisms underlying PFAS-induced hepatocarcinogenesis. By utilizing high-throughput target prediction bioinformatic strategies and relevant toxicogenomic databases, we systematically identified HCC-specific targets of PFOA and PFOS, thereby overcoming the limitations of single-target experimental studies and enabling a more holistic understanding of PFAS-HCC interactions. Through network toxicology analysis and machine learning-based prognostic modeling across six independent cohorts, we not only identified core PFAS-related prognostic targets but also established their potential prognostic significance in HCC. This model’s predictive power was validated by consistently high AUC values at various time points (1, 3, and 5 years) in different cohorts, underscoring its reliability in distinguishing high-risk and low-risk patients. In addition, the incorporation of single-cell RNA sequencing and spatial transcriptomic analyses allowed the dissection of cell type-specific and spatial expression patterns of the identified targets within the heterogeneous TME. These findings provide insights into how PFAS may exert their biological effects not only on malignant cells but also on non-malignant stromal and immune cell populations. Additionally, molecular docking simulations were employed to validate the structural binding affinities between PFAS compounds and core target proteins, thereby reinforcing the biological plausibility of the predicted interactions.

Notably, each of the six core targets plays a distinct role in HCC. APOA1 is associated with longer OS among HCC patients and has been shown to regulate immune responses. Elevated levels of APOA1 correlate with a decrease in inflammatory markers, potentially slowing HCC progression by modulating the activation of immune cells, including macrophages and T cells, while also reducing cytokine production [42]. Research further indicates that APOA1 overexpression can impair HCC cell growth and inhibit proliferation through lipid metabolism regulation [43, 44]. ESR1 activates the expression of genes involved in inhibiting cancer cell proliferation and inducing apoptosis. By activating downstream pathways, such as the p38 MAPK pathway, ESR1 helps limit the migration and invasion of HCC cells, which are critical factors in tumor metastasis [45, 46]. We also observed that IGF1 is highly expressed in macrophages, although its role in HCC progression remains unclear. However, in the context of wound healing and tissue repair, IGF1 enhances macrophage survival and function at injury sites, thus contributing to faster tissue regeneration by promoting cell proliferation and collagen synthesis [47, 48]. This may be associated with extracellular matrix (ECM) formation by malignant cells in HCC, providing a scaffold that supports tumor progression. PPARGC1A is expressed at low levels in HCC and is associated with poor prognosis. Wei et al. reported that PPARGC1A dysfunction caused by m6 A modification contributes to HCC progression and Lenvatinib resistance, which can be reversed by metformin [49]. The function of SERPINE1 in HCC remains largely unknown since only previous prognostic studies included this gene as a candidate for their prediction models or pan-cancer analysis. Xu Z et al. conducted a pan-cancer analysis concerning SERPINE1 and revealed that aberrant expression of SERPINE1 is common in cancers and is associated with poor patient prognosis, impaired cancer immunity, immunotherapy and chemotherapy resistance [50–53]. PON1 has been shown to exhibit antioxidant properties, playing a crucial role in reducing oxidative stress. It can hydrolyze lipid peroxides, thus protecting cells from oxidative damage. This activity is important in preventing cardiovascular and neurodegenerative diseases, where oxidative stress plays a key role [54, 55]. Collectively, these six core targets play significant roles in HCC oncogenesis, progression, and prognosis.

While this study provides new insights into the molecular mechanisms by which PFAS exposure contributes to HCC, an important next step is to explore potential therapeutic interventions targeting the identified core genes and their associated pathways. Several of these targets are already implicated in lipid metabolism, inflammation, and carcinogenesis, making them attractive candidates for drug development. For example, the anti-tumor effects of SERPINE1 inhibitors have been explored in various cancers, and SERPINE1 inhibitors may constitute a viable therapeutic strategy for PFAS-induced HCC. Additionally, targeting PPARGC1A, a key regulator of mitochondrial biogenesis and energy metabolism, with PPAR agonists such as fenofibrate could mitigate PFAS-induced metabolic reprogramming in liver cancer cells. Future research should focus on screening small-molecule inhibitors and natural compounds that modulate these targets to assess their potential efficacy in reducing PFAS-related hepatotoxicity and tumor progression [56].

In addition to therapeutic strategies, the findings of this study also have important policy implications. Given the increasing evidence linking PFAS exposure to liver toxicity and carcinogenesis, regulatory actions must be strengthened to limit human exposure. Policies such as stricter drinking water limits, bans on high-risk PFAS compounds, and enhanced industrial waste regulations could significantly reduce environmental contamination [57]. Additionally, biomonitoring programs should be expanded to assess longitudinal PFAS exposure levels in at-risk populations, particularly those living near contaminated water sources or industrial sites [58, 59]. Investment in remediation technologies, such as activated carbon filtration and advanced oxidation processes, may further mitigate PFAS contamination in water supplies and food systems.

Despite our efforts to integrate multi-omics approaches and computational toxicology to investigate PFAS exposure in HCC, this study has several limitations that warrant attention in future research. First, although transcriptional changes in PFAS-related target genes were confirmed via RT-qPCR and IHC staining, this method primarily reflects gene expression alterations at the mRNA and protein levels and does not capture the full spectrum of downstream functional consequences or regulatory complexity. Additional layers of molecular regulation, such as post-translational modifications, epigenetic alterations, and pathway activation, remain unexplored. Future studies may incorporate phosphoproteomic and epigenomic profiling to elucidate these mechanisms and provide a more holistic view of PFAS-induced hepatocarcinogenesis. Furthermore, this study is limited by its reliance on publicly available datasets and predictive computational models, and direct real-world validation of PFAS exposure is lacking. Although epidemiological studies have suggested potential links between PFAS and HCC, long-term, low-dose exposure data, particularly with quantifiable PFAS levels in human tissues or blood, are scarce. Large-scale prospective cohort studies incorporating direct PFAS measurements, individual metabolic variability, and cumulative exposure effects would significantly increase the translational relevance of the findings.

To address these gaps, future research should explore how PFAS exposure influences fundamental cellular processes in HCC through in vitro and in vivo experiments. Functional assays assessing cell proliferation, apoptosis, migration, invasion, and metabolic activity can be employed to characterize the phenotypic effects of PFAS. Oxidative stress levels may be measured via reactive oxygen species (ROS) detection, while lipid accumulation may be measured via Oil Red O staining, and mitochondrial health may be measured through mitochondrial membrane potential assays or electron microscopy. In addition, functional studies targeting the core genes identified in this study, through RNA interference (siRNA) or CRISPR-Cas9-mediated gene editing, could help determine whether transcript-level changes translate into functional phenotypes. Beyond the cellular level, in vivo models, such as chronic low-dose PFAS exposure in mice, could better mimic human environmental conditions and enable assessment of liver injury, fibrosis, immune infiltration, and tumor progression. Comprehensive evaluation through histopathology, serum biomarkers, and immune profiling will further clarify the systemic impacts of PFAS exposure. Together, these follow-up studies can provide mechanistic insights into the role of PFAS in liver carcinogenesis and facilitate the development of effective diagnostic and therapeutic strategies.

## Conclusion

This study integrates multi-omics and computational toxicology to explore the role of PFAS exposure in HCC. We identified six key target genes (APOA1, ESR1, IGF1, PPARGC1A, SERPINE1, and PON1) and highlighted metabolic, immune, and oncogenic pathways involved in PFAS-induced hepatocarcinogenesis. Our PFASRHSig risk model showed high predictive accuracy for patient survival, and molecular docking confirmed strong PFAS-protein interactions. While these findings increase the understanding of PFAS toxicity, further experimental validation and real-world exposure studies are needed. This study provides new biomarkers, risk assessment tools, and potential therapeutic targets, emphasizing the urgent need for regulatory policies to mitigate PFAS-related health risks.

## Supplementary Information

Supplementary Material 1: Table S1. The 855 targets for PFOA and PFOS were collected from the CTD and SwissTargetPrediction databases. Table S2. The 965 targets highly relevant to HCC were identified via data from the TCGA-HCC cohort. Table S3. Results of the PPI analysis via Cytoscape. Table S4. Results of the KEGG enrichment analysis. Table S5. Top 3 terms of each functional module according to the MCODE algorithm. Table S6. The primer sequences of the candidate genes subjected to RT-qPCR

## Data Availability

All datasets analyzed in this study are publicly accessible from reputable repositories. The chemical structures and canonical SMILES representations of perfluorooctanoic acid (PFOA) and perfluorooctane sulfonate (PFOS) were obtained from the PubChem database (https://pubchem.ncbi.nlm.nih.gov). PFAS-related targets were identified via SwissTargetPrediction (http://www.swisstargetprediction.ch) and the Comparative Toxicogenomics Database (CTD, https://ctdbase.org). All protein information was standardized via the UniProt database (https://www.uniprot.org). The Venn diagram was constructed via an online bioinformatics tool (http://bioinformatics.psb.ugent.be/webtools/Venn/). Protein–protein interaction (PPI) networks were constructed via the STRING database (http://string-db.org), and functional enrichment analyses were conducted via Metascape (http://metascape.org). All network visualizations were generated via Cytoscape (version 3.10.2). Bulk RNA-seq datasets were retrieved from the TCGA-LIHC cohort via the UCSC Xena portal, the LICA and LIRI cohorts from the ICGC data portal, the CHCC cohort from the NODE database, and GSE14520 and GSE54236 from the GEO database. Single-cell RNA-seq data (GSE149614) were obtained from the GEO database, while spatial transcriptomics data were retrieved from the National Genomics Data Center (NGDC) under accession number HRA000437. Processed data files used in downstream analyses, including gene expression matrices, risk scores, and enrichment results, are provided in the supplementary materials. Clinical validation data (RT-qPCR and IHC) generated from patient tissue samples are available from the corresponding author upon reasonable request, in accordance with the approved institutional ethical guidelines.

## Code availability

All source codes used in this study are publicly available at the following GitHub repository: https://github.com/StevenJiajunli/PFAS-HCC. The repository contains scripts for PFASRHSig establishment and assessment, single-cell landscape construction, expression pattern exploration and additional functions for visualization.
